# Torsion of Atypical Meckel's Diverticulum Treated by Laparoscopic-Assisted Surgery

**DOI:** 10.1155/2017/4514829

**Published:** 2017-07-13

**Authors:** Atsushi Kohga, Kimihiro Yamashita, Yuto Hasegawa, Kiyoshige Yajima, Takuya Okumura, Jun Isogaki, Kenji Suzuki, Akihiro Kawabe, Akira Komiyama

**Affiliations:** ^1^Division of Surgery, Fujinomiya City General Hospital, Fujinomiya, Japan; ^2^Division of Pathology, Fujinomiya City General Hospital, Fujinomiya, Japan

## Abstract

**Introduction:**

Meckel's diverticulum (MD) is the most common congenital anomaly of the intestine, with an incidence of 2~4%. Of those, only 2% of patients with MD are symptomatic. Torsion of MD is extremely rare, and only a dozen cases have been previously reported.

**Case Report:**

The patient was a 49-year-old male who presented to our emergency room with a chief complaint of lower abdominal pain. Computed tomography imaging revealed an irregular polycystic mass connected to the small intestine that measured 7.5 cm in a diameter. A laparoscopic-assisted partial resection of the jejunum was performed. The lesion was found to have caused torsion and was located 130 cm from the ileocecal valve. The specimen was polycystic in appearance and showed communicating links with the submucosal layer of jejunum but not with the lumen. The pathological diagnosis was a torsion of an atypical presentation of MD.

**Conclusion:**

This case was different from typical cases of MD in that it was located on significantly oral side and had the appearance of polycystic morphology.

## 1. Introduction

Meckel's diverticulum (MD) is vestigial remnant of the vitelline duct and results from incomplete obliteration of the vitelline duct [1–3], the most common congenital anomaly of the intestine, with an incidence of 2~4% [2, 3]. By the 5th to 7th week of gestation, the yolk sac has been reduced to a slim stalk, as the vitelline duct. Incomplete obliteration of the vitelline duct results in MD [2, 3]. Typical MD contains all the layers of intestine, because the yolk sac is continuous with the embryo and contains all the layers of intestinal wall [3]. Most cases of MD are asymptomatic, and only 4~16% of patients with MD become symptomatic [1, 3, 4]. The size of the diverticulum is associated with the complication rate [5], and a variety of complications have been reported [2, 3]. Of these, torsion of MD is an extremely rare clinical condition. Here, we report an extremely rare case of an atypical MD that macroscopically mimicked the extraintestinal growth of a small intestinal tumour and was treated by laparoscopic-assisted surgery.

## 2. Case Report

The patient was a 49-year-old male who presented to our emergency department with the complaint of a 4-day duration of lower abdominal pain. A physical examination revealed tenderness to palpation of the lower abdomen without signs of peritoneal irritation. There was no previous history of comorbid medical or surgical illness except for a recurrent feeling of abdominal discomfort that had persisted for several years. Laboratory data showed slight leukocytosis (WBC 112 × 10^2^/*μ*L) with elevated C-reactive protein (CRP 30.67 mg/dl).

**Figure 1:**
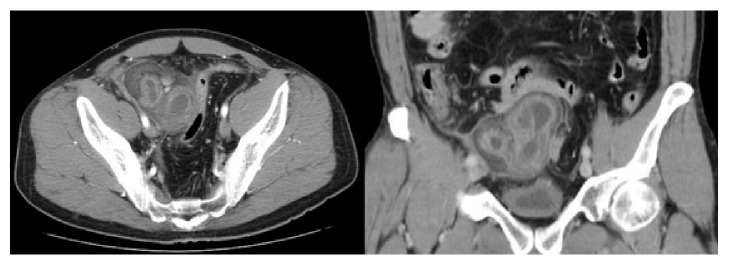
A computed tomography (CT) image revealed irregular polycystic mass connected to small intestine measuring 7.5 cm in a diameter in the lower abdomen.

**Figure 2:**
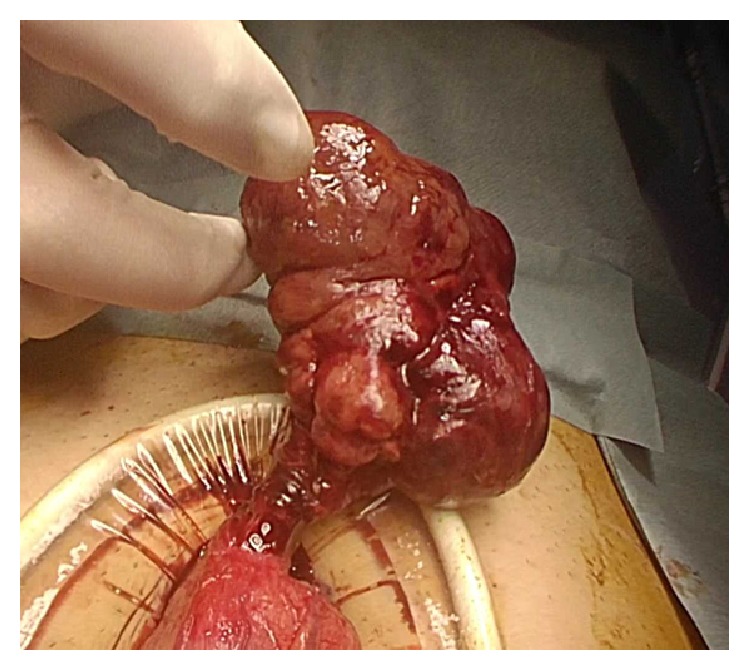
The lesion was found to cause torsion and congested.

**Figure 3:**
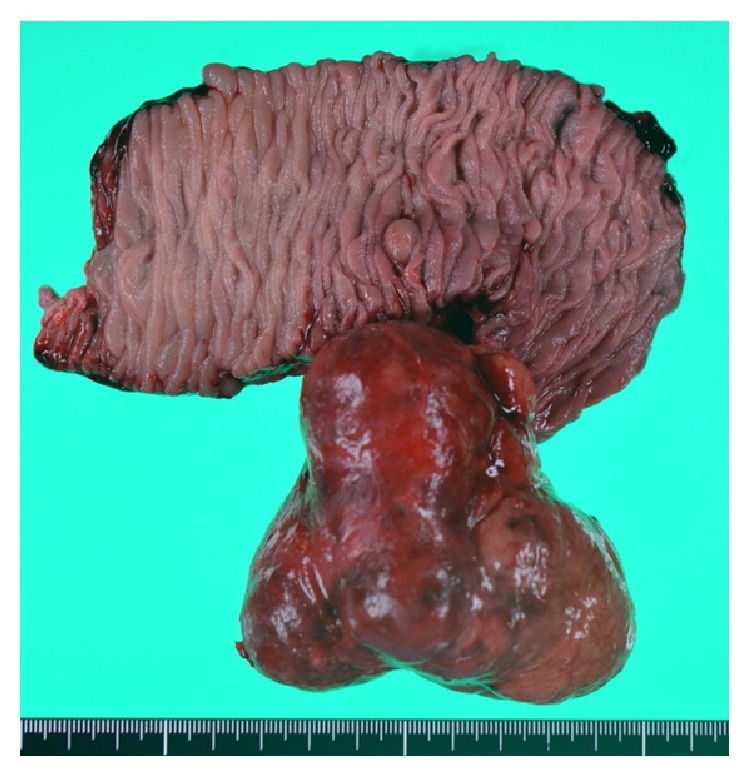
Partial jejunum resection including the lesion was performed. The specimen was solid irregular mass that looks like extraintestinal growth tumour.

**Figure 4:**
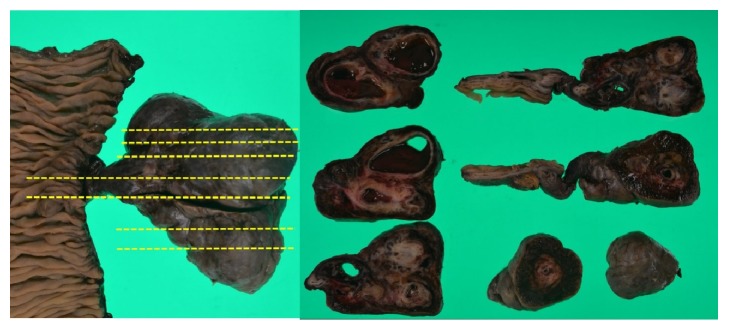
Macroscopically, irregular polycystic appearance was found on the cut surface containing fluid.

**Figure 5:**
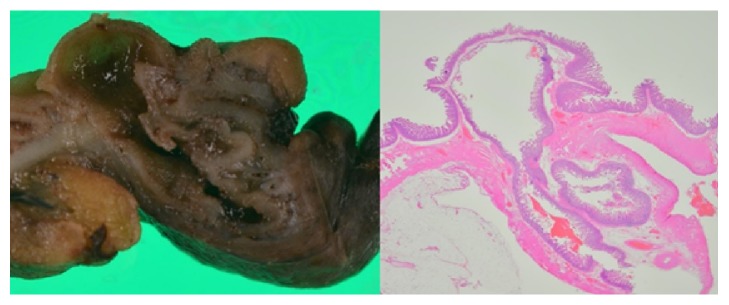
One of the cysts showed fistulation with submucosal layer of small intestine. Communicating with the lumen was not apparent.

**Figure 6:**
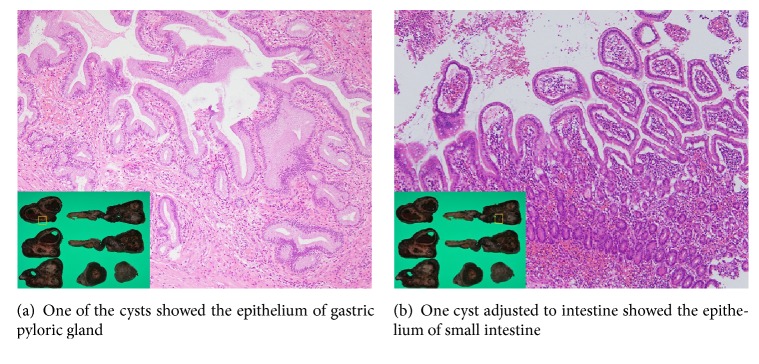


A computed tomography (CT) image revealed an irregular polycystic mass that was connected to the small intestine and measured 7.5 cm in diameter in the lower abdomen (Figure 1). The preoperative diagnosis was either a massive Meckel's diverticulum or a small intestinal tumour. We planned to perform laparoscopic-assisted surgery. During the laparoscopy, the lesion was found to have caused torsion and congested. After dividing the inflammatory adhesions, the lesion was removed through the umbilical incision (Figure 2). We confirmed that the lesion was located 130 cm from the ileocecal valve and 50 cm from the ligament of Treitz. Partial resection of the jejunum, which included the lesion, was performed. The specimen was a solid irregular mass that looked like an extraintestinal growth tumour with a narrow neck (Figures 2 and 3). Macroscopically, an irregular polycystic appearance was observed on the cut surface, and the mass was fluid-containing. One of the cysts showed fistulation with the submucosal layer of small intestine; however, communicating links with the lumen were not apparent (Figures 4 and 5). Microscopically, the inner surfaces of the cysts were covered with layers of epithelium. One of the cysts exhibited the epithelium of the gastric pyloric gland, while another cyst adjacent to the intestine exhibited the epithelium of the small intestine (Figures 6(a) and 6(b)). The lesion was diagnosed as atypical MD with torsion. The postoperative course of treatment was uneventful, and the patient was discharged on the 11th postoperative day without postoperative complications.

## 3. Discussion

In this report, we present an extremely rare case of atypical MD with torsion. This case differs from typical MD in that it was located significantly on oral side and had an atypical morphological appearance. First, regarding the location, the lesion in this case was located 130 cm from ileocecal valve and was 50 cm from the ligament of Treitz. Generally, MD is located on the antimesenteric border of the intestine and is usually found within 100 cm of the ileocecal valve [6, 7]. However, MD has been reported up to 180 cm from the ileocecal valve [7]. Therefore, the lesion in this case was located significantly on oral side compared with typical MD.

Second, in terms of morphology, this case looked like an extraintestinal growth tumour with a narrow neck. This was apparently different from typical MD. The present case showed an absence of communicating links with the lumen of the adjacent bowel and an irregular polycystic appearance. These finding are not seen in typical MD. Previously, several cases have been reported to be diagnosed with MD without communication links with the adjacent lumen [8–11]. One hypothesis of this disruption is that the links of communication had been obliterated as a result of a developmental or an inflammatory process [10, 11]. In the present, case, the polycystic appearance might have also been caused by obliteration of the consecutive lumen as a result of a developmental process or chronic inflammation. Pathologically, the present case showed ectopic gastric mucosa, which is consistent with the diagnosis of MD. In MD, heterotopic tissues such as gastric mucosa, duodenal mucosa, jejunal mucosa, and pancreatic tissue are present in 30 to 65% [5]. Of these, more than 60% are gastric mucosa [6, 12].

Regarding the differential diagnosis, we had to rule out intestinal duplication. In the present case, an antimesenteric location is grounds for exclusion of intestinal duplication.

Generally, most cases of MD are asymptomatic, and only 4~16% of patients with MD become symptomatic [1, 3, 4]. Symptoms include gastrointestinal bleeding, intestinal obstruction, diverticulitis, intussusception, and perforation [1–4]. By contrast, torsion of MD is extremely rare, and only a dozen cases have been previously reported in the English-language literature [13–20] (Table 1). Diverticular length and base diameter are important factors associated with torsion. An elongated variant with a narrow neck, like the lesion seen in the present case, may be a risk factor for torsion [13, 17]. In the present case, the patient had recurrent feelings of abdominal discomfort that had persisted for several years. This history suggested that recurrent torsion of the lesion had been occurring throughout that period. Chronic inflammation caused by recurrent torsion might result in a characteristic morphology that includes a polycystic appearance.

Regarding treatment, surgical resection is the standard treatment for symptomatic MD [2, 3]. In the present case, we performed laparoscopic-assisted surgery. Recent reports have suggested that laparoscopic-assisted surgery, including single incision laparoscopic-assisted surgery (SILS), is feasible for patients with symptomatic MD [21–23]. In the present case, we did not attempt SILS because the lesion was of considerable size and we could not preoperatively rule out the possibility of a small intestinal tumour. However, we believe that SILS is a feasible procedure for typical cases of symptomatic MD, including MD with torsion, but only if the preoperative diagnosis of MD was made and if the lesion was relatively small enough to pass through the umbilical incision. To our knowledge, no cases of MD with torsion treated by laparoscopic-assisted surgery have previously been reported.

## 4. Conclusions

We reported an extremely rare case of atypical MD with torsion. This case was different from typical cases of MD in that it was located on significantly oral side and had the appearance of polycystic morphology.

## Figures and Tables

**Table 1:** Previous reports of Meckel's diverticulum torsion in English literature.

Number	Author	Year	Age	Sex	Symptoms	Operative procedure	Size of MD/ Distance from ICV
(1)	Tan and Zheng	2005	51	Male	Abdominal pain Vomiting	Small bowel resection	10 × 3/60 cm
(2)	Limas et al.	2006	6	Male	Abdominal pain Fever, nausea	Diverticulectomy	16 × 4/50 cm
(3)	Kiyak et al.	2009	42	Male	Abdominal pain	Diverticulectomy	7.5 × 1.5/80 cm
(4)	Murruste et al.	2014	41	Male	Abdominal pain Nausea	Small bowel resection	12 × 14/50 cm
(5)	Tenreiro et al.	2015	18	Male	Abdominal pain Fever, vomiting	Small bowel resection	10 × 2/50 cm
(6)	Payá-Llorente	2015	67	Male	Abdominal pain Distention	Small bowel resection	17/NA cm
(7)	Ren et al.	2015	23	Female	Abdominal pain Nausea	Small bowel resection	8 × 3/60 cm
(8)	Kirmizi et al.	2016	32	Male	Abdominal pain Fever, vomiting	Small bowel resection	12 × 5/90 cm
(9)	Our case	2017	49	Male	Abdominal pain	LA small bowel resection	8 × 7.5/130 cm

MD: Meckel's diverticulum; ICV: ileocecal valve; LA: laparoscopic-assisted; NA: not announced.
